# Exploration and validation of a novel prognostic signature based on comprehensive bioinformatics analysis in hepatocellular carcinoma

**DOI:** 10.1042/BSR20203263

**Published:** 2020-11-13

**Authors:** Xiaofei Wang, Jie Qiao, Rongqi Wang

**Affiliations:** 1 Department of Oncology Surgery, Beijing Traditional Chinese Medicine Hospital, Capital Medical University, Beijing 100010, China; 2 Department of Traditional and Western Medical Hepatology, Third Hospital of Hebei Medical University, Shijiazhuang 050051, China

**Keywords:** Hepatocellular Carcinoma, Nomogram, Prognostic factors, Survival, WGCNA

## Abstract

The present study aimed to construct a novel signature for indicating the prognostic outcomes of hepatocellular carcinoma (HCC). Gene expression profiles were downloaded from Gene Expression Omnibus (GEO), The Cancer Genome Atlas (TCGA) and the International Cancer Genome Consortium (ICGC) databases. The prognosis-related genes with differential expression were identified with weighted gene co-expression network analysis (WGCNA), univariate analysis, the least absolute shrinkage and selection operator (LASSO). With the stepwise regression analysis, a risk score was constructed based on the expression levels of five genes: Risk score = (−0.7736* CCNB2) + (1.0083* DYNC1LI1) + (−0.6755* KIF11) + (0.9588* SPC25) + (1.5237* KIF18A), which can be applied as a signature for predicting the prognosis of HCC patients. The prediction capacity of the risk score for overall survival was validated with both TCGA and ICGC cohorts. The 1-, 3- and 5-year ROC curves were plotted, in which the AUC was 0.842, 0.726 and 0.699 in TCGA cohort and 0.734, 0.691 and 0.700 in ICGC cohort, respectively. Moreover, the expression levels of the five genes were determined in clinical tumor and normal specimens with immunohistochemistry. The novel signature has exhibited good prediction efficacy for the overall survival of HCC patients.

## Introduction

Hepatocellular carcinoma (HCC) has been one of the most prevalent cancers in the world with very poor prognosis [1]. Despite recent advances in surgical treatment and immunotherapy, the survival rates remain very low (with 5-year survival of approximately 17%) [2]. There was a particularly high risk of HCC in China due to the infection of hepatitis virus, especially hepatitis B virus (HBV) [3]. The heterogeneity among HCC patients has been recognized and molecular genetic methods have revealed different subgroups associated with distinct overall outcomes.

Identification of reliable diagnostic and prognostic biomarkers is critical for HCC treatment. It is well known that serum alpha-fetoprotein (AFP) was the most widely used biomarker for HCC diagnosis and prognostic [4,5]. TNM (tumor-node-metastasis) stage system was still commonly used to help predict HCC prognosis [6]. With the rapid development in genome-sequencing technologies and bioinformatic algorithms, many molecular signatures and genetic markers have been identified to improve the prognosis prediction in HCC patients [7,8]. For instance, Moeini et al. identified an immune-related gene expression pattern including 172 genes in early-stage HCC patients that was associated with risk of HCC development in patients with cirrhosis [9]. Zhang et al. established a 14-gene signature for predicting HCC outcome and recurrence [10]. Pinyol et al. discovered a 146-gene signature for improving sorafenib’s effectiveness in treating HCC [11]. Deep learning from genomic data has been an effective way of excavating prognostic signatures to promote precise treatment of HCC.

In the present study, we first identified robust differentially expressed genes (DEGs) as seed genes in the protein–protein network from four datasets. Then genes interacting with DEGs were extracted from String database. Weighted Gene Co-expression Network Analysis (WGCNA) was performed to screen survival-related module and ten hub genes from protein–protein network were selected to explore the prognostic signature. Univariate, Lasso Cox and stepwise regression analyses were used to identify novel prognostic biomarkers and construct the model in TCGA cohort. The model was further validated in an external cohort LIRI-JP from ICGC database. Nomogram was also established and the calibration curves showed it was able to predict survival accurately. Gene ontology (GO), Kyoto Encyclopedia of Genes and Genomes (KEGG) and Gene Set Enrichment Analysis (GSEA) analyses revealed different functions and pathways enriched in high- and low-risk patients, respectively. In brief, the novel signature exhibited good prognostic prediction efficacy in the overall survival of HCC patients.

## Materials and methods

### Data resources

Four datasets were included in this analysis. GSE39791 and GSE57957 datasets were downloaded from the Gene Expression Omnibus (GEO) database (www.ncbi.nlm.nih.gov/geo/), including 72 and 39 pairs of tumor and matched non-tumor tissues. TCGA-LIHC dataset and clinical information were downloaded from The Cancer Genome Atlas (cancergenome.nih.gov) [12], involving 50 normal samples and 374 tumor samples. LIRI-JP dataset was downloaded from the International Cancer Genome Consortium (ICGC) database (www.icgc.org), including 202 normal samples and 243 tumor samples.

### Data processing and normalization

GEO datasets expression data were acquired from microarray platforms, while the expression data of TCGA and LIRI-JP were normalized RNA sequencing data (FPKM). For GSE39791 and GSE57957, the matrix was filtered and normalized with Limma R package. The batch effect between TCGA and ICGC datasets was removed with SVA R package. All calculations were performed on log2-transformed expression values. TCGA dataset was applied as training cohort and 329 patients with overall survival (OS) ranged from 91 to 3675 days were included. ICGC was applied as external validation set including 232 patients with OS ranged from 90 to 2160 days.

### Differentially expressed genes (DEGs)

Differential genes expression analysis was performed with the Limma R package in GSE39791 and GSE57957, and with Wilcox Test in TCGA and ICGC datasets. DEGs were screened with cut-off criteria set as false discovery ratio (FDR) <0.05 and absolute fold change >1. These DEGs were considered as seed genes in the following protein–protein network.

### Protein genes interacting with DEGs

Protein network data (scored links between proteins) was downloaded from STRING database (https://string-db.org/). Common genes interacting with DEGs were identified with R 3.6.1 in TCGA and ICGC datasets.

### Weighted Gene Co-expression Network Analysis (WGCNA)

Protein genes interacting with DEGs were mapped and used to construct Weighted Gene Co-expression Network Analysis (WGCNA) in TCGA dataset with WGCNA R package. Survival-related module was carried out through the biggest Pearson correlation and the most significant *P*-value both with survival time and status.

### Survival-related genes identification in TCGA dataset

For the genes involved in the survival-related module from WGCNA, the univariate Cox regression analysis was performed by the R Survival package to identify the candidate survival-related genes with a univariate *P*-value < 0.05. Gene functional analyses were performed via the GO and KEGG pathways enrichments with clusterProfiler R package. A protein–protein interaction (PPI) network was then generated via STRING v11.0 (string-db.org). Hub prognostic genes ranked top ten were screened by Cytohubba plugin with Cytoscape v3.7.2.

### Developing and validation of prognostic signature

With the identified ten prognostic genes, the least absolute shrinkage and selection operator (LASSO) and Stepwise regression analyses were performed to explore the prognostic signature in TCGA which was further validated in ICGC. Patients were divided into high- and low-risk groups according to the median risk score of TCGA. Kaplan–Meier (KM) survival and receiver operating characteristic (ROC) curves were plotted by survival and survivalROC R packages. The areas under curves (AUCs) were used to describe accuracy and performance.

### Independent prognostic prediction analysis

Multivariate independent prognostic analyses were applied with survival R package in both TCGA and ICGC cohorts. Age, grade, gender, stage, TMN (tumor, metastasis, node) and Risk score were included in TCGA. Gender, age, stage, prior malignancy and Risk score were included in ICGC. A total of 214 patients in TCGA cohort and 228 patients in ICGC cohort with complete clinical information were included. HR was calculated and expressed with forest plot. Five-year receiver operating characteristic curves (ROC) of risk score, age, stage, TMN (tumor, metastasis, node) were plotted with survivalROC R package. AUC of the ROC curve was also provided.

### Construction of the nomogram

According to the multivariate independent prognostic analysis results of TCGA cohort, a prognostic nomogram including significant factors (*P*-value<0.05) was developed and constructed with rms R package. The receiver operating characteristic and 5-year calibration curves were also plotted.

### Gene Set Enrichment Analysis (GSEA)

In order to investigate the differences of biological functions and pathways between high- and low-risk patients, Gene set enrichment analysis was performed using GSEA software (v 4.0.1). The most significantly enriched Kyoto Encyclopedia of Genes and Genomes (KEGG) pathways were plotted.

### Survival analysis and Immunohistochemistry

The survival analysis of prognosis genes in the signature was performed with KM Plotter (http://www.kmplot.com/). Patients were divided into high- and low-expression groups according to the median levels. For the purpose of revealing the different expressions between tumor and normal tissues, immunohistochemical experiments were carried out. A total of 20 HCC and matched adjacent normal tissues were collected from the Third Hospital of Hebei Medical University. Tissues were fixed with 4% neutral formaldehyde for 24 h. About 4-μm sections were generated after dehydration and paraffin embedding. After dewaxing in xylene and rehydrating through a graded series of ethanol, the antigen retrieval was performed via the wet autoclaving method in the presence of citrate buffer for 3 min. After cooling, 0.3% hydrogen peroxide was used to block nonspecific antigens for 30 min. Tissue slides were washed in PBS-T three times and blocked in 10% fetal bovine serum for 20 min. Incubation with primary antibodies including (rabbit-anti CCNB2, 1:200, Sigma), (rabbit-anti DYNC1L1, 1:2500, Sigma), (rabbit-anti KIF11, 1:200, Sigma), (rabbit-anti SPC25, 1:200, Sigma) and (rabbit-anti KIF18A, 1:200, Sigma) was conducted overnight at 4°C. PBS without primary antibody was used as the negative control. When the incubation was over, tissue slides were rewarmed at room temperature for 40 min and incubated with secondary antibodies at 37°C water bath for 20 min. DAB chromogenic reagent was applied to develop the stain and hematoxylin was used to stain the nucleus. The sections were finally dehydrated and mounted with a neutral resin onto slides. Integrated optical density (IOD) values were calculated and analyzed by Image Pro Plus 6.0 software. Paired *t*-test was used to compare the differences between the tumor and normal tissues.

## Results

### Identification of DEGs between normal and tumor tissues

**Figure 1:**
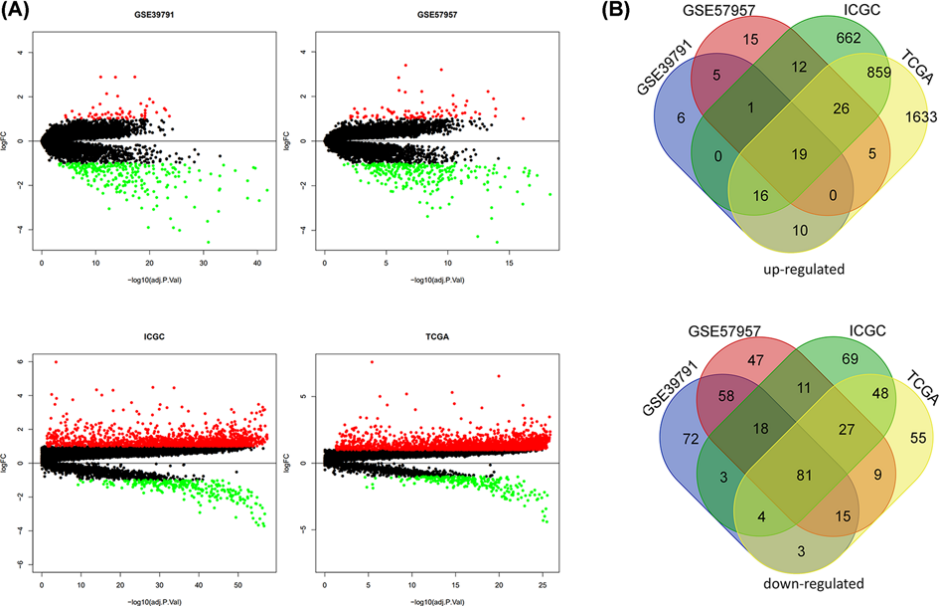
Identification of differentially expressed genes (DEGs) (**A**) Volcano plots of DEGs between normal and tumor tissues. (**B**) Venn diagram of common DEGs.

A total of 57 up-regulated and 254 down-regulated DEGs were obtained from GSE39791. For GSE57957, 83 up-regulated and 266 down-regulated DEGs were screened. There were 2568 up-regulated and 242 down-regulated DEGs identified in TCGA-LIHC dataset. For ICGC-LIRI-JP dataset, 1595 up-regulated and 261 down-regulated DEGs were identified. The distributions of DEGs were represented by volcano maps (Figure 1A). Finally, 100 DEGs were included in the overlapped DEGs identified from the four datasets, including 19 up-regulated and 81 down-regulated DEGs (Figure 1B). Then, the protein genes interacted with DEGs were identified in TCGA and ICGC datasets. A total of 3617 mRNAs were identified to interact with the 100 DEGs.

### WGCNA and key module

**Figure 2:**
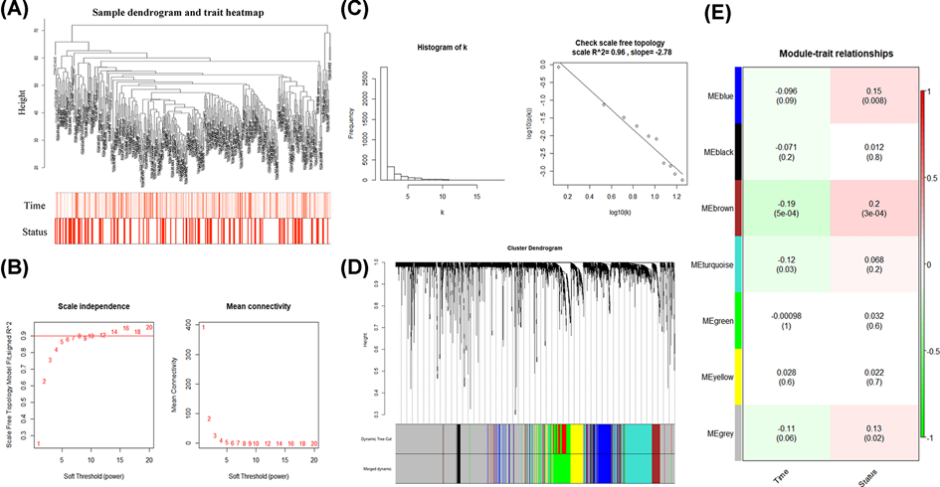
Key module identified by WGCNA (**A**) Sample dendrogram and clinical-traits heatmap. (**B**) Determination of soft-thresholding power. (**C**) Scale-free topology (*R*^2^ = 0.96) (**D**) Clustering dendrogram. Both the original and merged modules were presented. (**E**) Module-trait relationship were evaluated by correlations between module eigengenes and clinical traits.

The expression profiles of 3617 mRNAs in TCGA dataset were extracted for the construction of co-expression network by the WGCNA R package. A total of 329 samples with survival time and status in TCGA dataset were included. Four outlier samples were removed and sample dendrogram and clinical-traits heatmap was plotted (Figure 2A). The scale-free network was optimized with soft-threshold power (β) value determined as 8 (Figure 2B). The scale-free topology was plotted, with *R*^2^ = 0.96 and slope = −2.78 (Figure 2C). The distribution followed a straight line, indicating an approximately scale-free topology.

The co-expression modules were further generated with Dynamic tree cutting. The parameter was set as 0.25 to merge closely associated modules. A total of seven modules were obtained (Figure 2D). The relationships between each module and clinical traits were shown (Figure 2E). The brown module including 261 genes showed the highest Pearson’s correlation coefficient (PCC) and the most significant *P*-value for both survival time (PCC = −0.19, *P*=0.0005) and survival status (PCC = 0.2, *P*=0.0003) (Figure 2E).

### Identification of survival-related hub genes

**Figure 3:**
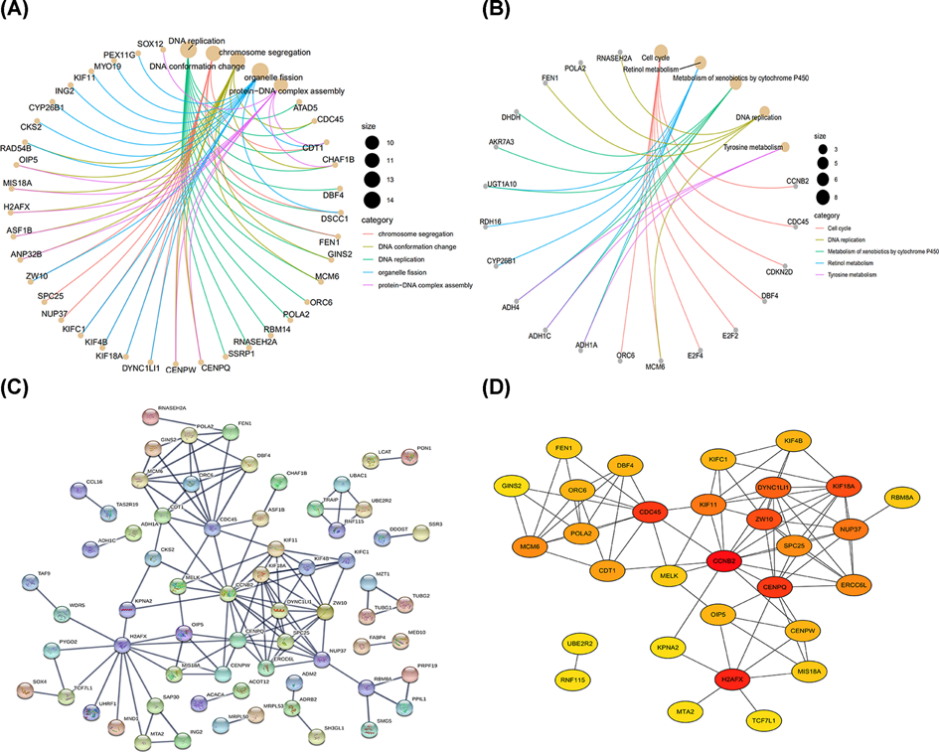
The analysis of survival-related hub genes Gene functional analyses of 187 involved genes were performed via the GO (**A**) and KEGG pathways (**B**) enrichments with clusterProfiler R package. The involved categories for GO analysis were chromosome segregation, DNA conformation change, DNA replication, organelle fission and protein–DNA complex assembly. The involved KEGG terms were cell cycle, DNA replication, metabolism of xenobiotics by cytochrome P450, retinol metabolism and tyrosine metabolism. The size indicated the number of genes enriched in each term. (**C**) The protein–protein interaction (PPI) network was constructed via STRING v11.0 (string-db.org). (**D**) Top 30 prognostic genes screened by Cytohubba plugin with Cytoscape v3.7.2. Darker color indicated higher rank.

The survival-related genes in the brown module were further analyzed. First, the univariate Cox regression analysis was performed. A total of 187 genes were selected (*P*-value < 0.05). GO and KEGG pathways enrichments were performed on the 187 genes for exploring their potential biological functions. Results showed that DNA replication, chromosome segregation, DNA conformation change, organelle fission and protein–DNA complex assembly were the most enriched GO terms (Figure 3A), while cell cycle, retinol metabolism, metabolism of xenobiotics by cytochrome P450 and DNA replication were the most enriched KEGG terms (Figure 3B).

PPI network was generated for screening prognosis related hub genes (Figure 3C). Hub prognostic genes ranked top 30 were screened and plotted by Cytohubba plugin with Cytoscape v3.7.2 (Figure 3D). Some genes ranked high in the network, such as CDC45, CCNB2, CENPQ and H2AFX.

### Construction and validation of prognostic signature

**Figure 4:**
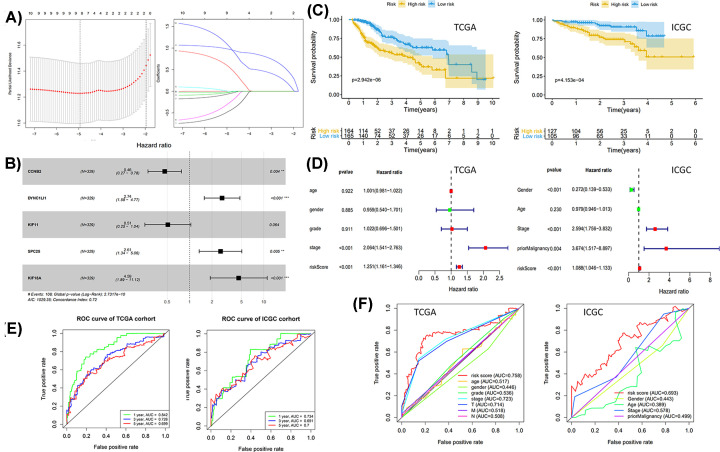
Construction and validation of prognostic signature (**A**) LASSO regression analysis. (Left) Selection of tuning parameter (lambda); (Right) Dynamic LASSO coefficient profiling. (**B**) Stepwise regression analysis. (**C**) Kaplan–Meier survival analysis. (**D**) Independent prognostic prediction analysis. (**E**) 1-, 3- and 5-year ROC curves of Risk score. (**F**) 5-year ROC curves of Risk score and other clinical features.

We used TCGA cohort as Training set (*n*=329) and ICGC cohort as external validation set (*n*=232). With the identified top ten prognostic genes (CCNB2, H2AFX, CENPQ, CDC45, ZW10, KIF18A, DYNC1LI1, NUP37, KIF11, SPC25), the LASSO regression analysis was performed. Finally, eight key genes were identified (Figure 4A). The stepwise regression analysis was further applied to construct the model for obtaining prognostic signature (Figure 4B). Five prognostic genes were finally included in the model for calculating the Risk score. The HRs and coefficients were provided (Figure 4B). The risk scores of patients were calculated using the following formula: Risk score = (−0.7736* CCNB2) + (1.0083* DYNC1LI1) + (−0.6755* KIF11) + (0.9588* SPC25) + (1.5237* KIF18A).

Kaplan–Meier survival analysis indicated the survival probability of patients in high-risk and low-risk groups (*P*<0.001, Figure 4C).

Multivariate survival analyses were applied to evaluate whether the Risk scores were independent prognostic factors irrespective of other clinical features. Age, grade, gender, stage, TMN (tumor, metastasis, node) and Risk score were included in TCGA cohort. Gender, age, stage, prior Malignancy and Risk score were included in ICGC cohort. A total of 214 patients in TCGA cohort and 228 patients in ICGC cohort with complete clinical information were included. The Hazard ratio was plotted (Figure 4D). The Risk score was verified as independent prognostic predictor.

1-, 3- and 5-year ROC curves of Risk score were also plotted, with the AUC of 0.842, 0.726 and 0.699 in TCGA cohort and 0.734, 0.691 and 0.700 in ICGC cohort, respectively (Figure 4E). The AUC value indicated good prognostic prediction efficacy.

The 5-year receiver operating characteristic curves (ROC) of Risk score and other clinical features were plotted with survivalROC R package. Results indicated that the Risk score provided a higher value of AUC compared to that of any other clinical features (Figure 4F).

**Figure 5:**
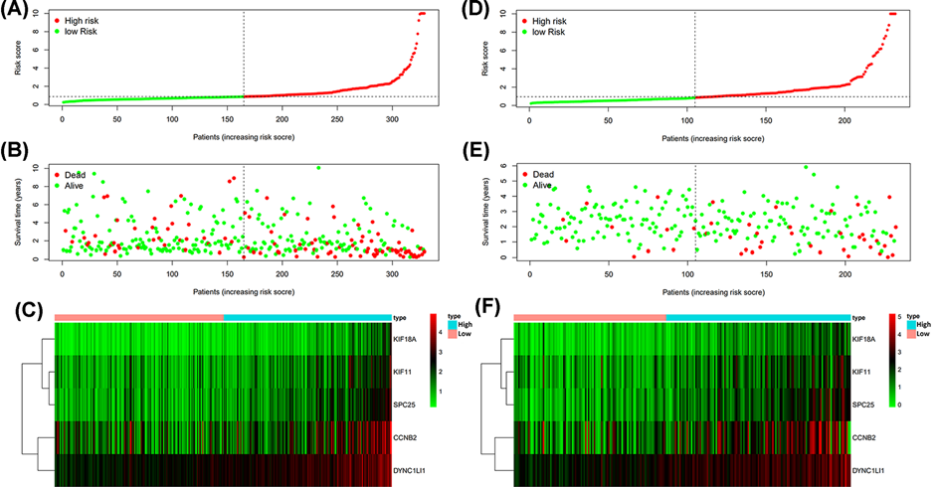
Analysis of prognosis signature in TCGA and ICGC cohorts (**A**) Distribution of patients’ risk score in TCGA. (**B**) Relationship of Risk score and Survival time in TCGA. (**C**) Heatmap of expression profiles of included genes in high and low risk groups in TCGA**.** (**D**) Distribution of patients’ risk score in ICGC. (**E**) Relationship of Risk score and Survival time in ICGC. (**F**) Heatmap of expression profiles of included genes in high and low risk groups in ICGC**.**

The distributions of patients’ survival status, risk score and the patterns of the prognosis signature in TCGA cohort (Figure 5A–C) and ICGC (Figure 5D–F) cohort were visualized.

### Construction of the nomogram

**Figure 6:**
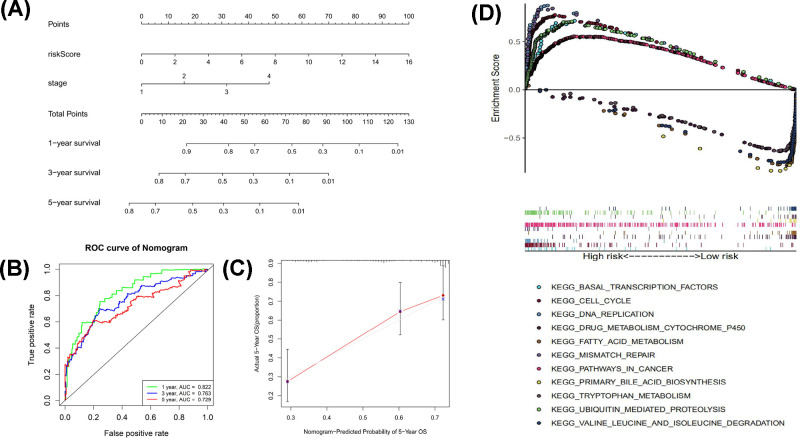
Construction of nomogram and GSEA (**A**) The nomogram of 1-, 3- and 5-year survival of TCGA cohort. (**B**) 1-, 3- and 5-year ROC curves of the nomogram. (**C**) The calibration curves of 5-year survival. The blue dot and light grey line represented the ideal prediction model, and the black solid line represented the observed model. (**D**) The most significantly enriched KEGG pathways between high- and low-risk patients.

According to the multivariate independent prognostic analysis results of TCGA cohort, a prognostic nomogram including Stage and Risk score (*P*-value<0.05) was developed and constructed with rms R package (Figure 6A). 1-, 3- and 5-year ROC curves of the nomogram were plotted, with the AUC of 0.822, 0.763 and 0.729 (Figure 6B). The AUC value indicated good prognostic prediction efficacy. The calibration curves were also plotted and indicated good prognostic prediction efficacy (Figure 6C).

### Gene set enrichment analysis (GSEA)

In order to investigate the differences of biological functions and pathways between high- and low-risk patients, Gene set enrichment analysis was performed using GSEA software (v 4.0.1). The most significantly enriched Kyoto Encyclopedia of Genes and Genomes (KEGG) pathways were plotted (Figure 6C). Results showed that DNA replication, Ubiquitin mediated proteolysis, mismatch repair, cell cycle, basal transcription factors, pathways in cancer were significantly enriched in the high-risk patients and primary bile acid biosynthesis, Fatty acid metabolism, valine leucine and isoleucine degradation, drug metabolism cytochrome P450 and tryptophan metabolism were the most significantly enriched in the low-risk patients (Figure 6C).

### Survival analysis and immunohistochemistry

**Figure 7:**
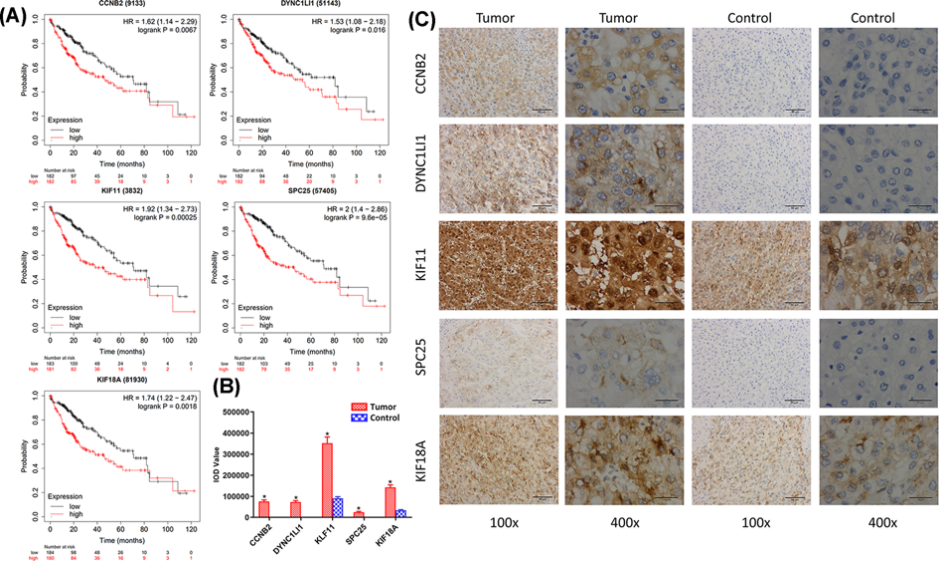
Kaplan–Meier survival curves and Immunohistochemistry Analysis (**A**) Overall survival analysis of the five prognosis genes from the KM plotter database. (**B**) IOD values in tumor and normal tissues. (**C**) Representative images of IHC in tumor and normal tissues at 100× (left) and 400× (right).

Kaplan–Meier survival curves of HCC patients with low and high expression of the five prognosis genes were generated by Kaplan–Meier Plotter (www.kmplot.com/). Results showed that all the five genes were associated with unfavorable prognosis with *P*-value<0.05 (Figure 7A). Immunohistochemistry data confirmed significant higher expression of the five gene proteins in HCC tissues with *P*-value<0.05 (Figure 7B). In tumor tissues, CCNB2, DYNC1LI1, SPC25 and KIF18A expressions were mainly detected in the cytoplasm, and KIF11 expression was detected mainly in the cytoplasm and nucleus (Figure 7C). In normal tissues, CCNB2, DYNCLI1 and SPC25 expressions were not detected. Medium expressions of KIF11 and KIF18A were found in normal tissues (Figure 7C).

## Discussion

With the rapid development in next-generation sequencing, our understanding of transcriptional alterations in HCC has been greatly improved. The heterogeneity among HCC patients has been recognized and molecular genetic methods have revealed different subgroups associated with distinct overall patient outcomes [13–15]. According to the survival prediction of patients with prognostic biomarkers, individualized treatment can be better developed in clinical practice [16,17]. In comparison with a single gene, a multigene-based model was more robust and precise in diagnosis and prognosis in many cancers [18,19].

In our study, a novel signature was identified to stratify HCC patients according to the risk score. We integrated multiple genes into a single signature via Lasso-Cox modeling. The novel signature was verified to be a significant predictor both in TCGA and ICGC cohorts. The five genes included in our signature were CCNB2, DYNC1LI1, KIF11, SPC25 and KIF18A. The heatmaps were plotted to reveal the risk of identified genes based on gene expression profile. As shown in the heatmaps, the expression of the five genes increased together with the risk score in both cohorts.

CCNB2, encoding Cyclin B2, is a key member of the cyclin family and plays an important role in cell cycle. Li et al. found that CCNB2 overexpression in HCC was associated with poor prognosis, and knockdown of CCNB2 could inhibit cell proliferation and migration, promote cell apoptosis, and lead to S phase arrest in HCC cell lines [20]. Wang et al. identified key prognostic genes including CCNB2 in HCC by integrated bioinformatics analysis [21]. DYNC1LI1, short for Cytoplasmic dynein 1 light intermediate chain 1, is one non-catalytic accessory component of the cytoplasmic dynein 1 complex and may play a certain role in binding dynein to membranous organelles or chromosomes. Even et al. found that depletion of DYNC1LI1 caused reduced proliferation, enhanced apoptosis rates, disrupted mitotic spindle assembly and G2/M arrest in cell cycle progression [22]. Chang et al. found that DYNC1LI1 expression was significantly associated with different types of mucin expression levels and could be used to predict the chemotherapeutic efficiency in colorectal cancer [23]. KIF11, short for kinesin family member 11, plays a role in chromosome positioning, centrosome separation and establishing a bipolar spindle during cell mitosis [24]. Jin et al. found high expression of KIF11 predicted a unfavorable prognosis in clear cell renal cell carcinoma [25]. Zhou et al. also found KIF11 functioned as an oncogene and was associated with poor outcomes in breast cancer patients [26]. Chen et al. found KIF11 overexpression was significantly associated with shorter relapse-free survival times in HCC [27]. SPC25 encodes a protein that may be involved in kinetochore–microtubule interaction and spindle checkpoint activity. Gu et al. screened 13 key genes including SPC25 in HCC by WGCNA [28]. Guo et al. identified SPC25 as a prognostic biomarker of HCC via random-forest algorithm [29]. Chen et al. revealed that SPC25 knockdown resulted in a significant decrease in proliferation and metastasis of HCC cells and increased protein levels of components of the p53 pathway *in vitro* [30]. KIF18A, is a member of the kinesin superfamily of microtubule-associated molecular motors. Janssen et al. found the loss of KIF18A resulted in a loss of tension across a subset of kinetochores accompanying spindle assembly checkpoint activation [31]. Luo et al. discovered that KIF18A promoted proliferation, invasion and metastasis of HCC cells by promoting the cell cycle signaling pathway [32]. Liao et al. found high KIF18A expression correlated with unfavorable prognosis in HCC [33]. These findings proved that the five screened genes played important roles in the development and progression of HCC.

However, there are also limitations in our study. It is mainly a retrospective analysis based on different databases and the functions of all the identified genes need to be explored in experiments. On the other hand, recent studies on the five genes involved in the signature have been very few, which may increase the uncertainties of their prognosis significances. In brief, we identified and reliably validated a novel signature which exhibited good prognostic prediction efficacy in the overall survival of HCC patients.

The present study established a signature for calculating the Risk score based on the expression levels of five genes, which predicted the overall survival of HCC patients. The present study provided an innovative analysis route for obtaining significant biomarkers. Several advantages were summarized, including large sample size from multiple databases and the combined analysis of PPI and WGCNA. Through comprehensive data analysis, the reliable result with good repeatability can be obtained, which can be verified with external data and clinical outcomes.

## Data Availability

All data, models, or code used in this study are available from the corresponding author by request.

## Abbreviations

DEG: differentially expressed gene
GEO: Gene Expression Omnibus
HCC: hepatocellular carcinoma
ICGC: International Cancer Genome Consortium
LASSO: least absolute shrinkage and selection operator
PCC: Pearson’s correlation coefficient
ROC: receiver operating characteristic curves
TCGA: The Cancer Genome Atlas
WGCNA: weighted gene co-expression network analysis
